# Physiology of Menstruation

**Published:** 1875-08

**Authors:** W. G. Drake

**Affiliations:** Atlanta, Ga.


PHYSIOLOGY OF MENSTRUATION.—As this subject is exciting considerable interest at the present time, we are glad of the opportunity to present to our readers the following letter from our friend, Dr. W. G. Drake, of Atlanta:

ATLANTA, GA., July 3, 1875.

*Messrs. Editors:*

In looking over the recent proceedings of the Atlanta Academy of Medicine, I notice that Dr. J. M. Johnson states that he "does not believe that the ovaries have any connection with menstruation."

Several years ago, while practising in Alabama, I delivered a patient, from whom in about two weeks afterwards I removed the uterus entire and the follopian tubes. She made a good recovery, and, several months afterwards, as her general health improved, there was, at regular monthly intervals, a flow of blood per vaginam, corresponding in quantity to her former catamenia. If now the ovaries have no connection with menstruation, whence this flow? why this periodicity?

I will state further, to show that the ovaries must have been left intact, and had something to do with this periodical flow, she informed me that the orgasm experienced during coition was as exquisitely enjoyable as at any time previous to her impregnation. I have the specimen in my office.

Respectfully, W. G. Drake, M.D.

In order to any profitable discussion of the subject of menstruation and its causes, it is necessary that some definite understanding shall first be had as to the significancy of the term menstruation. The term is used by writers who attach to it widely differing meanings. If menstruation may occur, upon the one hand, without the uterus, as contended by some, and upon the other hand without the ovaries, as held by others, and especially in the absence of both uterus and ovaries, as in the case of Sarah A. Colcord, reported by Dr. Horatio R. Storer, of Boston, the latitude given to the term divests it of all its heretofore accepted significancy. With so wide a scope given it the term may be applied to the sanguineous discharge of infants at the breast as well as to the decrepid great-grandmother of ninety, just tottering into her grave. But it is not our purpose to discuss the subject at all at present. We design simply to put upon record the interesting and valuable observation of Dr. Drake for future reference and use.

